# Fetal Alcohol Spectrum Disorders – Diagnostic Difficulties in The Neonatal Period and New Diagnostic Approaches

**DOI:** 10.34763/devperiodmed.20192301.6066

**Published:** 2019-04-08

**Authors:** Iwona Jańczewska, Jolanta Wierzba, Monika Cichoń-Kotek, Alicja Jańczewska

**Affiliations:** 1 Department of Neonatology, Medical University of Gdansk, Gdansk Poland; 2 Department of General Nursery, Medical University of Gdansk, Gdansk Poland; 3 Department of Pediatrics, Hematology and Oncology, Medical University of Gdansk, Gdansk Poland; 4 Student of Medical University of Gdansk, Gdansk Poland

**Keywords:** FASD, Fetal Alcohol Spectrum Disorders, pregnancy, prenatal alcohol exposure, congenital abnormalities, fetal growth retardation, neonate, alcohol biomarkers, fatty acids, meconium, FASD, płodowy zespół alkoholowy, ciąża, etanol, wady wrodzone, opóźnienie wzrastania płodu

## Abstract

Fetal alcohol spectrum disorders (FASD) is a group of disorders that can occur in children whose mothers consumed alcohol in pregnancy.

Diagnosis of fetal alcohol syndrome is based on the appearance of growth deficiency, the presence of the three key features of facial dysmorphism (short palpebral fissures, thin upper lip, smooth or flattend philtrum) and/or disorders in the central nervous system (minimum 3) and prenatal exposure to alcohol (confirmed if possible).

Early diagnosis of fetal alcohol syndrome – after birth or in infancy – is very often impossible or very difficult due to the incomplete manifestation of the key dysmorphic features. The latest reports offer the chance of diagnosing children in the neonatal period.

The research focuses on the analysis of ethanol metabolites in the biological tissues in pregnant women or newborns. These unique ethanol metabolites include: fatty acid ethyl esters (FAEE) present in the meconium, blood, hair of the mother and the newborn, ethyl glucuronide in the placenta and meconium, urine, nails and hair, and phosphatidyl ethanol (PEth) found in the infant blood. The presence of fatty acid ethyl esters in the meconium could be a non-invasive and cost-effective method of early detection of disorders associated with prenatal alcohol exposure.

## Streszczenie

Spektrum płodowych zaburzeń alkoholowych to grupa objawów, które mogą wystąpić u dzieci, których matki spożywały alkohol w trakcie ciąży.

Diagnoza płodowego zespołu alkoholowego opiera się na stwierdzeniu zaburzeń wzrastania, obecności trzech kluczowych cech dysmorfii twarzy (krótkie szpary powiekowe, cienka warga górna, płaskie filtrum), obecności zaburzeń w funkcjonowaniu ośrodkowego układu nerwowego (minimum 3) oraz w miarę możliwości potwierdzonej prenatalnej ekspozycji na alkohol.

Bardzo często wczesna diagnoza płodowego zespołu alkoholowego po urodzeniu lub w wieku niemowlęcym jest niemożliwa albo bardzo utrudniona z uwagi na niepełną manifestację kluczowych cech amorficznych. Najnowsze doniesienia dają możliwość wstępnego diagnozowania już dzieci w wieku noworodkowym. Badania koncentrują się na analizie metabolitów etanolu w tkankach biologicznych u ciężarnej lub noworodków. Te unikalne metabolity etanolu obejmują: estry etylowe kwasów tłuszczowych (FAEE) obecne w smółce, krwi, włosach matki i noworodka, glukuronid etylu w łożysku i smółce, moczu, paznokciach i włosach oraz fosfatydyloetanolu (PEth) pobrane z krwi niemowląt. Obecność estrów etylowych kwasów tłuszczowych mogłaby być w przyszłości nieinwazyjną i opłacalną metodą wczesnego wykrywania zaburzeń związanych z prenatalną ekspozycją na alkohol.

## Introduction

According to the general definition, Fetal alcohol syndrome (FAS) is the collection of somatic, cognitive and behavioural disorders encountered in children exposed to alcohol during pregnancy.

The syndrome consists of: prenatal and postnatal growth retardation, central nervous system disorders and (mostly craniofacial) dysmorphic features.

The definition of this syndrome has changed over the years, and up to now physicians and scientists are aware that prenatal alcohol exposure (PAE) is associated with a spectrum of abnormalities, referred to as fetal alcohol spectrum disorders (FASD) rather than FAS [1, 2, 3, 4].

The physical, behavioural and learning disabilities, observed in FAS/FASD have lifelong implications.

Many guidelines have been published [1, 2, 3, 5, 6] since the first description of FAS. The majority of authors agreed that diagnosis would be easier if the symptoms were fully expressed and the prenatal alcohol exposure objectively confirmed [6, 7]. Early diagnosis makes it possible to implement proper therapeutic procedures.

## The prevalence of FASD

Previous studies estimated the prevalence of FAS in the general population of the United States at 0.2 – 1.5 per 1000 children, depending on the populations studied and the surveillance methods used [2]. Based on past in-school studies of various populations the frequency of FAS was estimated to be 2-7 per 1000 children in South Africa, Italy and USA [8]. May et al. have recently evaluated the prevalence estimates for the entire continuum of FASD range to be 24-48 per 1000 individuals (2.4% - 4.8%) among first grade children in a representative Midwestern US city [9]. These data are close to the findings from European studies, in which the rate of FASD ranged from 20.3 to 40.5 per 1000 children in a province in Italy and 40.77 per 1000 in Croatia [10, 11].

FASD is estimated as the leading cause of mental retardation [12].

## Abnormal outcomes of pregnancy

Alcohol intake in pregnant women has important implications for pregnancy outcomes. As early as 1900 Sullivan reported an increased rate of spontaneous abortions and stillbirths among women heavily abusing alcohol. Prenatal alcohol exposure is associated with placental dysfunction, decreased placental size, impaired blood flow, nutrient transport and endocrine changes in pregnant women, any of which could play a role in stillbirth and preterm deliveries. Many authors suggested that maternal alcohol abuse leads to significantly higher incidence of premature placental separation, stillbirth, spontaneous abortion and premature birth [13, 14, 15].

## The effects of prenatal alcohol exposure on the central nervous system

Brain damage is the most significant effect in the cohort of patients affected by prenatal alcohol exposure (PAE). Ethanol can induce cell damage, leading to structural anomalies of grey and/or white matter. Studies involving animal models have shown that prenatal alcohol exposure affects all stages of brain development from neurogenesis to myelination, through a variety of mechanisms, including disrupted cell-cell interactions, altered gene expression, oxidative stress, and growth factor signalling disruptions. The consequences of such central nervous system developmental defects include: reduction in the overall brain volumes in children and adolescents causing microcephaly, agenesis or malformation of the corpus callosum, ventriculomegaly, small cerebellum, hippocampus abnormalities and a variety of other brain abnormalities due to neuronal and glial migration errors. It has been shown that maternal ethanol exposure is associated with cortical thickness in a number of brain regions in FASD individuals [16, 17, 18, 19].

## Dysmorphic features of FASD

So far it has not been possible to describe one pathognomonic dysmorphic feature in FASD. The characteristic facial features of FAS were first described by Jones and Smith in 1973, later redefined by Astley, Clarren, clarified by Hoyme et al. in 2005 and updated in 2016 [3, 5, 6]. The most distinctive facial characteristics are: hypertelorism, short palpebral fissures, smooth philtrum, and a thin upper vermillion border, a relatively short-upturned nose, midface hypoplasia, micrognathia. Moreover, you can observe ptosis, epicanthic folds, hypoplasia of nails, limbs and palmar crease abnormalities and the abnormal position or formation of the ears [7]. All of these characteristics may also be present in other genetic syndromes. In view of the above, FAS facial criteria have been restricted to 3 anomalies: short palpebral fissures (at or below the 3rd percentile), smooth or flattened philtrum (4 or 5 on the 5-point Likert scale of the lip-philtrum guide) and a thin vermilion border of the upper lip (4 or 5 on the 5-point Likert scale of the lip-philtrum guide) [5].

The presentation of individual features may be variably expressed with age and tends to improve with the advancement in age of the affected individuals. Only 7% of the children are diagnosed in the first days of life, the average age of diagnosis is about 3.3 years [4, 20, 21]. The patients’ facial features can change when they get older, therefore the diagnosis should be based on the point in time when these features were most severely expressed [5].

## FASD characteristics in the neonatal period

Several detailed scales for FAS/FASD recognition were established. FASD and other fetal alcohol effects, including alcohol-related neurodevelopmental disorder (ARND) are diagnosed with the presence of some CNS (Central Nervous System) anomalies and behavioural or cognitive abnormalities. These criteria are appropriate for the evaluation of older children, when it is possible to assess the neurodevelopmental pattern [3, 5, 6].

However, in the neonatal period, when the potential neurological, cognitive, and behavioural effects are not apparent, significant difficulties in making the diagnosis can occur. Therefore, these components should be eliminated from the diagnostic criteria for newborns.

## Microsomy

The presence of the main facial features and growth retardation is thought to be most significant for FAS/ FASD recognition in the neonatal period [22, 23]. Alcohol-exposed neonates can be small for gestational age and remain below average throughout their lives with respect to head circumference, weight and height. Animal and human studies have found an association between high alcohol consumption during the first trimester of pregnancy and the facial features and presence of intrauterine growth retardation (IUGR) in the affected children [24].

Although there are many other causes of IUGR, this symptom is significant for diagnosis when there is a history of maternal alcohol drinking during pregnancy [25]. Jacobson et al. (1994) found lower birth weight (509 g less than average) and shorter length (4 cm shorter than average) in the neonates born to women who drank heavily, i.e. at least an average of 60 ml absolute alcohol per day during pregnancy, compared to the neonates of women who did not drink during pregnancy [26]. Other studies have also indicated that heavy alcohol consumption during pregnancy was associated with an increased risk for low birth weight (<2500 g), birth length and head circumference below the 10th percentile [27]. These prenatal growth deficits have been shown to persist through infancy [25].

Feldman et al. analysed the associations with the prenatal alcohol exposure pattern and occurrence of the 3 characteristic facial features (short palpebral fissures, smooth philtrum, thin vermillion border) and growth deficiencies on birth weight, birth length, and head circumference. These studies have shown that the number of binge episodes and the number of average alcoholic beverages per day during the first trimester were the highest risk-exposure pattern for alcohol-related facial features and growth deficiencies. These associations were linear, without evidence of a threshold [20].

If there is no alcohol exposure in the third trimester, the growth parameters can be normal. Some maternal pregnancy complications, such as gestational diabetes, can lead to increased fetal size and can mask the effects of growth retardation caused by prenatal alcohol exposure.

## Microcephaly

Microcephaly is also a significant component of the diagnostic criteria in the newborn period [20, 23, 27]. Consistently with previous studies, May has shown an association between maternal drinking, especially binge episodes and decreased head circumference and facial dysmorphism [28].

## Facial characteristics

With some limitations, craniofacial abnormalities can already be seen in the neonatal period. The palpebral fissure length is difficult to measure due to the very frequent swelling of the eyes after birth, and also to the fact that most newborn infants do not open their eyes long enough to make the measurement possible [23]. In addition, midface hypoplasia can be typical for some ethnic groups. Regarding these difficulties, researchers diagnosing FAS in neonates considered the following facial features: depressed or wide nasal bridge, anterverted nares, long or hypoplastic philtrum and thin upper vermillion. They suggest that a neonate should be diagnosed as having FAS/FASD, if the infant presented at least four out of six of these facial features [22].

## Birth defects

The congenital anomalies of many other organs were associated with prenatal alcohol exposure. Some authors have linked maternal alcohol consumption with neural tube defects, oral clefts and the occurrence of the Pierre-Robin syndrome. It is thought that about one third of the children prenatally exposed to alcohol present a higher risk of cardiac anomalies, such as: Ventricular Septal Defect (VSD), Atrial Septal Defect (ASD), Coarctation of the Aorta (CoAo), Hypoplastic Aortic Arch (HAA), and conotruncal heart malformations. Binge drinking during the second month of pregnancy was associated with bilateral renal agenesis or hypoplasia. Hydroneprosis, or ureteral duplication can also be encountered. Neonates prenatally exposed to alcohol also presented with other malformations, particularly concerning the limbs, chest and skin. Skeletal anomalies include: sunken chest, pectus carinatum, flection contractures, radioulnar synostosis, syndactyly, camptodactyly and hypoplastic nails. The visible skin problems in FAS/FASD neonates are: simian palmer creases, raised red birthmarks (capillary hemangiomas), hirsutism. Some FAS/FASD neonates demonstrated a high incidence of strabismus, retinal vascular anomalies and conductive or neurosensory hearing loss [1,6, 29, 30,31].

## The differential diagnosis

While the classic “presentation” of FAS usually raises no diagnostic doubts, even the specialist may have difficulties when the phenotype is not complete or atypical [1]. The differential diagnosis of FAS/FASD in the dysmorphological and neurobehavioral aspects of FASD is broad. Many genetic and malformation syndromes have some of the clinical characteristics of FAS.

Children prenatally exposed to other embryotoxic factors, like hydantoin, valproic acid, toluene or high levels of phenylalanine in maternal phenylketonuria (PKU) present with common similar facial characteristics, impaired growth, birth defects, developmental and behavioural anomalies.

For example, the fetal valproate syndrome is characterized, among others, by a long upper lip with relatively shallow philtrum, a relatively small mouth with downturned angles, and a thin upper vermilion border. Pre- and postnatal growth deficiency, short nose, microcephaly, epicantic folds, hypertelorysm, ptosis and developmental delay are often described in fetal hydantoin syndrome. In pregnant women with PKU (phenyloketonuria), noncompliance can result in maternal PKU syndrome, where high phenylalanine (Phe) levels cause severe fetal complications. Children born with maternal PKU syndrome suffer from microcephaly, craniofacial dysmorphism, low birth weight, congenital heart disease, developmental delays, and mental retardation [32].

Abusing illegal drugs by pregnant women (e.g. cocaine and others) may cause characteristics similar to those observed in infants exposed to alcohol, such as intrauterine growth retardation, smaller head circumference and behavioural and cognitive deficits [26].

Many genetic syndromes share some common characteristics with FAS, namely: Noonan syndrome, Williams syndrome, blepharophimosis syndrome (BPES), Cornelia de Lange syndrome, velocardiofacial syndrome (VCFS), Dubowitz syndrome and Aarskog syndrome. Cytogenetic and molecular analysis is used to differentiate these diseases from FAS [33]. Many clinical experiences have demonstrated that FASD should always be a diagnosis of exclusion [6].

## Alcohol intake in pregnant women

Maternal alcohol consumption in pregnancy is an important public health problem. Unlike other harmful behaviours of pregnant women, such as smoking and taking illegal drugs, drinking alcohol during pregnancy is common across most social groups, irrespectively of age and educational status. The estimated percentage of women drinking heavily during pregnancy ranges from 2% to 13%, depending on the population sample studied, the definition “heavy” and the study method used [26, 34]. Data from the latest research estimating the proportion of women drinking alcohol during pregnancy in Europe showed that on average 15.8% of pregnant women reported alcohol consumption. The highest proportion of alcohol consumption during pregnancy was found in the UK (28.5%), Russia (26.5%), and Switzerland (20.9%) and the lowest in Norway (4.1%), Sweden (7.2%) and Poland (9.7%) [35].

## Self-reporting maternal alcohol use during pregnancy

Many cases of both FAS and partial FAS are missed in the newborn period. One of the many problems with the identification of babies at risk is not knowing which infants have been exposed, as many women underreport their use of alcohol during pregnancy. Individuals tend to deny to themselves and to others that they have this problem due to the social stigmatization of drinking in pregnancy, so obtaining an accurate estimate of how many pregnant women drink can be difficult. The use of maternal self-reports for collection of alcohol consumption data could result in underreporting or misreporting. Women who drank higher doses of alcohol during pregnancy, as evidenced by the ethanol metabolites found in the meconium samples of their infants rarely admitted to drinking alcohol during pregnancy. Self-reporting is likely to miss identifying some individuals at risk of PAE [36, 37]. May et al. (2013) found a high frequency of alcohol consumption during pregnancy, even regarding binge drinking (40% of the women surveyed). However, they admitted that the sample mothers were extraordinarily forthcoming and reliable in reporting alcohol use [28].

## Biomarkers of fetal alcohol exposure

Proper and early diagnosis of fetal alcohol effects in infants may be limited by the lack of pathognomonic presentation at birth and in early infancy. Many more children with prenatal exposure to alcohol are affected neurobehaviorally than the number who exhibit the structural features of FAS [4, 12]. The new approach to finding objective data of prenatal alcohol exposure focuses on analyzing ethanol metabolites in the biological tissues of either the pregnant woman or the neonate [38].

These unique ethanol metabolites include: fatty acid ethyl esters (FAEEs) present in the meconium, blood, the hair of the mother and the newborn, ethyl glucuronide in the placenta and meconium, urine, nails and hair [38, 39, 40]. Detection of frees in the meconium has been proposed as a screening method for prenatal alcohol exposure, revealing maternal drinking from about the 20th gestational week. When alcohol is metabolized, fatty acid ethyl esters (FAEEs) accumulate in the meconium, the first stool passed by the newborn. This non-oxidative metabolite can be a marker of the second and third trimester alcohol exposure. In Canada, 17 of the 682 (2.5%) meconium samples tested were found positive for high levels of exposure, which was 5 times greater than the number of high risk exposures detected via the questionnaire [38, 39]. Although more research needs to be done to establish a threshold for the meconium concentration of FAEEs, this test can be proposed as a cost-effective method of identifying high-risk offspring.

## Summary

Identification of alcohol-affected children continues to be challenging, especially during infancy, the optimal time to initiate remedial interventions. Early diagnosis of prenatal effects is needed because craniofacial dysmorphic features that characterize FASD are found only in a small proportion of children with alcohol-related neurobehavioral impairments and can be difficult to detect in infancy and childhood. Proposed testing of FAEE in meconium samples as a biomarker of prenatal ethanol exposure can facilitate the early diagnosis and intervention in the affected children. Introducing appropriate patient care is crucial to reach the best possible neurological development of the affected individuals, and leads to decreasing the risk for secondary disabilities encountered in FASD patients.
